# P02.177. Effects of Bach Rescue remedy on cardiac autonomic balance in healthy women

**DOI:** 10.1186/1472-6882-12-S1-P233

**Published:** 2012-06-12

**Authors:** S Yang, Y Wang

**Affiliations:** 1 Institute of Natural Healing Sciences, NanHua University, DaLin, ChiaYi, Taiwan

## Purpose

The Rescue remedy has been used as a stress relief formula by practitioners of Bach flower remedies. The objective of the present study is to evaluate the effect of Bach Rescue remedy on cardiac autonomic balance in healthy women.

## Methods

In this two-stage crossover trial, seven females (mean age 26±4 years) were randomlyassigned to a sequence of two treatments (Rescue remedy and placebo) with a one-month wash-out period. The Rescue remedy consisted of four drops of five flower essences (cherry plum, clematis, impatiens, rock rose and Star of Bethlehem) dissolved in brandy and 250 ml of distilled water. The placebo consisted of four drops of brandy in 250 ml of distilled water. Cardiac autonomic functions were evaluated by frequency domain indices (LF, low frequency power and HF, high frequency power) of heart rate variability at baseline and after the intervention. Percentage changes were calculated and compared between the Rescue remedy and placebo groups using Wilcoxon signed rank sum test.

## Results

Both the mean percentage changes of LnLF (natural logarithm-transformed LF) and LF/HF ratio were significantly different between the two groups. The mean percentage change of LnLF in the Rescue remedy group was -15.9+/-7.4% compared with 18.3+/-21.6% in the placebo group. The mean percentage change of LF/HF was -27.8+/-13.0% in the Rescue remedy group compared with 53.2+/-89.1% in the placebo group.

## Conclusion

This is the first study using a double-blind randomized crossover design to evaluate the effect of Bach Rescue remedy on heart rate variability in healthy women. Increased parasympathetic activity and decreased sympathetic activity were observed in individuals receiving Bach Rescue remedy. These changes may explain the stress relieving effect of Bach Rescue remedy.

